# HIV-1 Vpr Induces the Degradation of ZIP and sZIP, Adaptors of the NuRD Chromatin Remodeling Complex, by Hijacking DCAF1/VprBP

**DOI:** 10.1371/journal.pone.0077320

**Published:** 2013-10-08

**Authors:** Claire Maudet, Adèle Sourisce, Loïc Dragin, Hichem Lahouassa, Jean-Christophe Rain, Serge Bouaziz, Bertha Cécilia Ramirez, Florence Margottin-Goguet

**Affiliations:** 1 Institut National de la Sante et de la recherche Medicale Inserm U1016, Institut Cochin, Paris, France; 2 CNRS UMR8104, Paris, France; 3 University Paris Descartes, Paris, France; 4 Institute for Molecular Infection Biology, University of Würzburg, Würzburg, Germany; 5 HYBRIGENICS services SAS, Paris, France; 6 CNRS UMR8015, Paris, France; George Mason University, United States of America

## Abstract

The Vpr protein from type 1 and type 2 Human Immunodeficiency Viruses (HIV-1 and HIV-2) is thought to inactivate several host proteins through the hijacking of the DCAF1 adaptor of the Cul4A ubiquitin ligase. Here, we identified two transcriptional regulators, ZIP and sZIP, as Vpr-binding proteins degraded in the presence of Vpr. ZIP and sZIP have been shown to act through the recruitment of the NuRD chromatin remodeling complex. Strikingly, chromatin is the only cellular fraction where Vpr is present together with Cul4A ubiquitin ligase subunits. Components of the NuRD complex and exogenous ZIP and sZIP were also associated with this fraction. Several lines of evidence indicate that Vpr induces ZIP and sZIP degradation by hijacking DCAF1: (i) Vpr induced a drastic decrease of exogenously expressed ZIP and sZIP in a dose-dependent manner, (ii) this decrease relied on the proteasome activity, (iii) ZIP or sZIP degradation was impaired in the presence of a DCAF1-binding deficient Vpr mutant or when DCAF1 expression was silenced. Vpr-mediated ZIP and sZIP degradation did not correlate with the growth-related Vpr activities, namely G2 arrest and G2 arrest-independent cytotoxicity. Nonetheless, infection with HIV-1 viruses expressing Vpr led to the degradation of the two proteins. Altogether our results highlight the existence of two host transcription factors inactivated by Vpr. The role of Vpr-mediated ZIP and sZIP degradation in the HIV-1 replication cycle remains to be deciphered.

## Introduction

Vpr is a 96-amino acid protein encoded by both HIV-1 and HIV-2, which were cross-transmitted to humans from two distinct primate lentiviral lineages that naturally infect chimpanzees and sooty mangabeys respectively [1]. Vpr belongs to the set of so-called viral auxiliary proteins, which play a crucial role at the host-virus interface by inactivating host restriction factors. For example, Vif induces the degradation of APOBEC3G to avoid mutations in the viral DNA, Vpu inactivates tetherin/BST-2 to trigger virus release and Vpx inactivates SAMHD1 to increase the levels of dNTP, essential precursors of viral DNA synthesis [2-8]. However, the function of Vpr has remained elusive.

The presence of Vpr in the incoming virion argues for a role of this protein in the early steps of the viral life cycle, before *de novo* expression from integrated proviral DNA. Accordingly, an increase in HIV-1 transduction is observed in the presence of Vpr in macrophages and in dendritic cells [9-12]. Numerous activities have been ascribed to Vpr, including its ability to arrest dividing cells at the G2/M transition, to mediate a G2 arrest-independent cytotoxic effect, to activate transcription from LTR and cellular promoters, to increase the fidelity of reverse transcription or to induce the degradation of the UNG2 uracil DNA glycosylase (for reviews, see [13,14]). Among these properties, the most widely studied is its ability to arrest cell cycle progression at the G2 phase. We and others have described a mechanism in which Vpr connects the DCAF1 adaptor of the Cul4A ubiquitin ligase to a so far unidentified host target protein (hereafter referred to as the G2 target), which is required for the G2/M transition [15-21]. As a result, the Vpr target protein undergoes poly-ubiquitination and subsequent proteasome-mediated degradation, which precludes cell entry into mitosis. This cytostatic activity was shown to depend on entry into the S-phase and on the ability of Vpr to associate with chromatin [22-24]. In addition, Vpr may use DCAF1 to trigger a G2 arrest-independent cytotoxic effect and to induce the degradation of UNG2 [25-27]. Whether other Vpr activities depend on the recruitment of Cul4A is unknown. As described for Vif and Vpu, Vpr may use the same ubiquitin ligase to induce the degradation of several specific host proteins. Li et al. further proposed a model in which Vpr interaction with the hHR23A protein would be an additional step toward the targeting of Vpr substrates to the proteasome [28]. We now face the challenge of identifying these corresponding target proteins, which may represent negative cellular factors for viral growth.

The nucleosome remodeling complex Mi-2/NuRD plays a key role in various cellular processes such as transcriptional repression, cell cycle progression, chromatin assembly, DNA damage response and maintenance of genome integrity (for reviews, see [29-31]). It contains different protein subunits which assemble in a combinatorial manner, leading to different outcomes and cell-type specific functions. Core subunits with enzymatic activities are chromodomain-helicase-DNA-binding protein 3 (CHD3 or Mi2-α) and CHD4 (or Mi-2β) and histone deacetylase 1 and 2 (HDAC1 and HDAC2). In addition to their role within the NuRD complex, some subunits also interact with other complexes, such as RbAp46 (also named RBBP7) present in several chromatin modification complexes and HDAC1 and HDAC2 found in other transcriptional repressor complexes. The NuRD complex plays a role in normal developmental processes, for example at different stages of hematopoietic differentiation (reviewed in [31]). Its role in cancer progression is not well-defined since it can promote or suppress tumorigenesis depending on the context (reviewed in [30]). Transcription repression by the NuRD complex is usually mediated through its recruitment to gene promoters by a tissue-specific transcription factor, mostly an oncogene but sometimes a tumor suppressor. ZIP, also known as ZGPAT, is a Zn finger and G-patch domain-containing transcription repressor, which recruits the NuRD complex and inhibits cell proliferation and survival, while its isoform sZIP seems to exert an opposite effect [32,33].

Here, we identified ZIP and sZIP as direct interacting partners of HIV-1 Vpr. Both proteins are degraded in the presence of the viral protein via the hijacking of the DCAF1 ubiquitin ligase. Nonetheless Vpr-mediated ZIP or sZIP degradation does not explain the cytostatic or cytotoxic activities of Vpr.

## Results

### HIV-1 Vpr interacts with ZIP and sZIP

**Figure 1:**
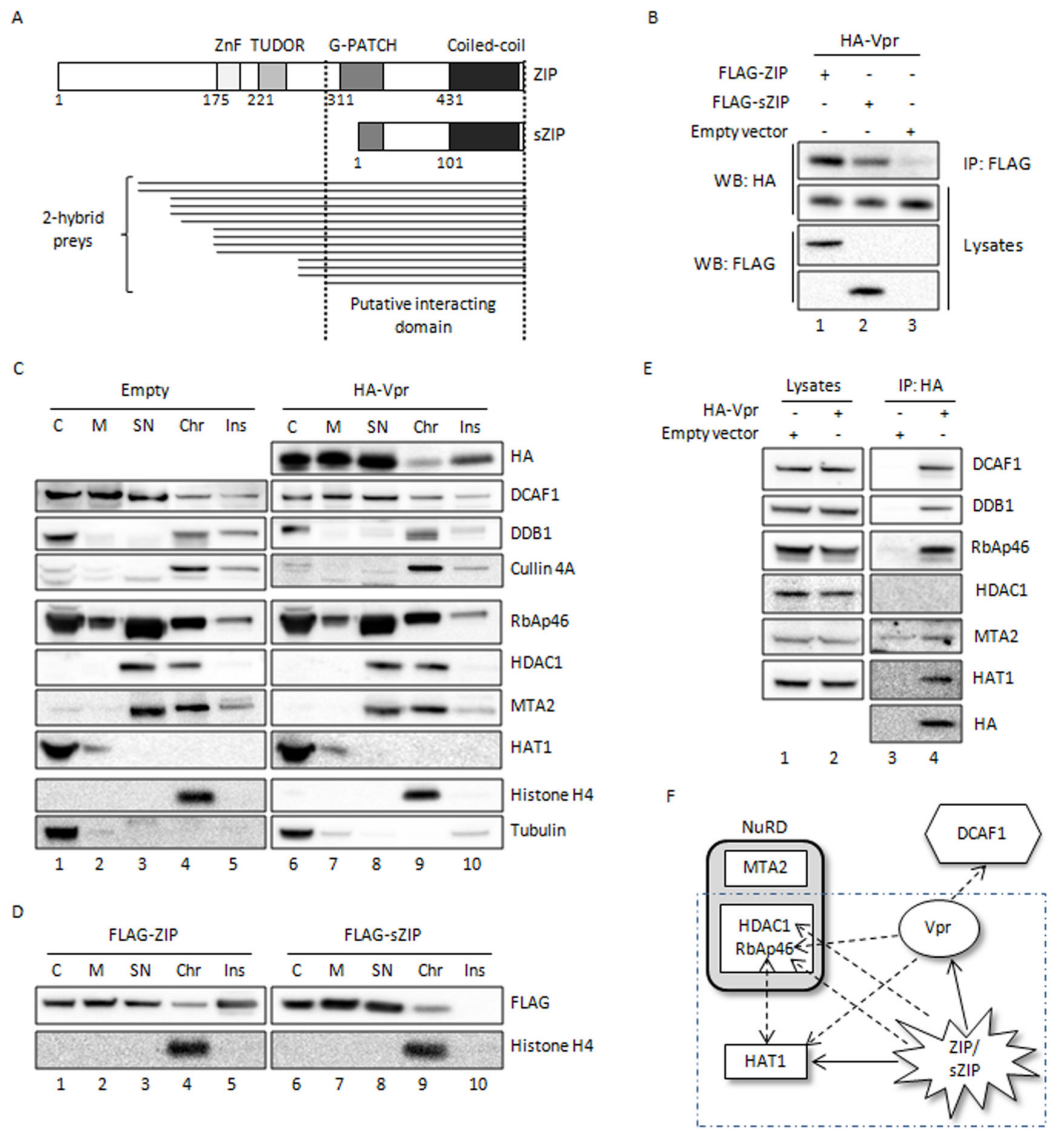
Interaction of HIV-1 Vpr with ZIP and sZIP and with the NuRD complex. **A**. Schema of ZIP and sZIP and Vpr-interacting fragments. Vpr was used as bait in a yeast two-hybrid screen of oligo d(T)-primed cDNAs from human CEMC7 cells. ZIP and sZIP isoforms are represented by boxes, with their known domains in different shades of grey. The preys matching with ZIP are drawn as thin lines below the diagram representing ZIP and sZIP proteins. **B**. Vpr interacts with both ZIP and sZIP in HEK293T cells. HEK293T cells were transfected with vectors expressing HA-tagged Vpr and the indicated FLAG-tagged proteins. Cell lysates were prepared 48h post-transfection and subjected to immunoprecipitation using anti-FLAG antibodies. After extensive washing, bound proteins were eluted from beads with a FLAG peptide. Immunoprecipitates (IP) and crude cell lysates (Lysates) were analyzed by Western blotting using the indicated antibodies. **C**. Chromatin is the only fraction where Vpr, Cul4A^DDB1^ and members of the Mi-2/NuRD complex (RbAp46, HDAC1 and MTA2) are detected together. HeLa cells were transfected with either a vector expressing HA-tagged Vpr or an empty vector. Cells were harvested 48h post-transfection and subcellular fractionation was performed on 2 10^6^ cells to obtain cytoplasmic (C), membrane (M), nuclear soluble (SN), chromatin-bound (Chr) and insoluble (Ins) protein extracts. The final volume ratio of each fraction is 2:2:1:1:1 respectively. The cellular distribution of the Vpr protein was analyzed by Western blot, as well as the cellular distribution of the indicated endogenous proteins. **D**. ZIP and sZIP are detected in the chromatin fraction. HeLa cells were transfected with vector expressing either FLAG-ZIP or FLAG-sZIP. Cells were harvested 48h post-transfection and subcellular fractionation was performed in the same conditions as described above. **E**. Vpr recruits RbAp46 and HAT1 in HEK293T cells. HEK293T cells were transfected with either a vector expressing HA-tagged Vpr or an empty vector. Cell lysates were prepared 48 h post-transfection and subjected to immunoprecipitation using anti-HA antibodies. Immunoprecipitates (IP) and crude cell lysates (Lysates) were analyzed by Western blotting using the indicated antibodies. **F**. Interactions detected between Vpr, ZIP/sZIP and the Mi-2/NuRD complex. Interactions detected by co-immunoprecipitation are represented on this diagram. The direction of the arrow indicates the direction of the co-immunoprecipitation (base of the arrow: immunoprecipitated protein, arrow: co-immunoprecipitated protein). Full arrows correspond to new interactions we have unraveled here and arrows in dotted line interactions previously described and confirmed in this study [32,33,38]. Interactions between ZIP/sZIP and HAT1, RbAp46 and HDAC1 are shown in Figure S2.

In order to identify new cellular partners of HIV-1 Vpr, an exhaustive yeast two-hybrid screen of cDNAs from human CEMC7 cells was performed using Vpr as bait. Among the proteins identified with multiple prey clones were known partners of Vpr, such as DCAF1, UNG2 or SAP145, and a new potential partner of Vpr, the transcriptional repressor ZIP. Alignment of prey insert sequences indicated that the domain interacting with Vpr was confined to the C-terminal region of ZIP (Figure 1A), a region shared by its sZIP isoform. Co-immunoprecipitation experiments in HEK293T cells confirmed the interaction between exogenously expressed Vpr and ZIP (Figure 1B, compare lane 1 and 3) and between Vpr and sZIP (Figure 1B, lane 2). Next, we asked whether Vpr and ZIP/sZIP could be present in the same cellular compartment, since on the one hand ZIP/sZIP associates with the NuRD chromatin remodeling complex [32,33] and on the other hand Vpr associates with chromatin [22,23]. We used a cellular fractionation assay that enables the separation of five distinct pools of proteins: cytoplasmic, membrane-bound, nuclear soluble, chromatin-bound and insoluble proteins. We validated the fractionation method by analyzing the localization of tubulin, present mostly in the cytoplasmic fraction (C), histone H4, found in the chromatin fraction (Chr), HDAC1 and MTA2 subunits of NuRD, present in the soluble fraction (SN) and in the chromatin-associated fraction (Chr) of the nucleus and finally RbAp46, detected in all the fractions [34-36]. Histone acetyltransferase 1 (HAT1), a previously characterized partner of RbAp46, was detected in the cytoplasm also confirming previous studies showing that this enzyme shuttles between the cytoplasm and the nucleus where it rapidly dissociates from histones [37]. Transfected HA-Vpr was found in the five distinct fractions, with about 2.5% of the total amount of the protein present in the chromatin-bound fraction (Figure 1C, HA panel, and Figure S1). The lack of valid antibodies for ZIP and sZIP led us to study the localization of the proteins expressed from transfected vectors. Both isoforms were found in all the fractions, except the insoluble fraction where only ZIP could be detected (Figure 1D). Strikingly the chromatin was the only compartment in which both Vpr and the Cul4A^DDB1^ ubiquitin ligase subunits (DCAF1, DDB1, and Cul4A) were present together (Figure 1C). Expression of Vpr did not significantly modify the cellular repartition of Cul4A^DDB1^ (Figure 1C) or the localization of exogenous ZIP or sZIP (data not shown). Next, we wondered whether Vpr was able to recruit the NuRD complex, as ZIP or sZIP does [32,33]. Vpr interacted with RbAp46, as well as with HAT1, confirming the data from Jäger et al., obtained from a global analysis of HIV interacting cellular partners [38] (Figure 1E). Of note, ZIP and sZIP were also able to recruit HAT1 in addition to subunits of the NuRD complex (Figure S2). However, Vpr did not interact with HDAC1 and interacted weakly with MTA-2 (Figure 1E). Our data, summarized on the scheme in Figure 1F, indicate that Vpr recruits RbAp46, HAT1, ZIP and sZIP, in addition to the Cul4A ubiquitin ligase.

### HIV-1 Vpr induces the degradation of ZIP and sZIP through the DCAF1 ubiquitin ligase

**Figure 2:**
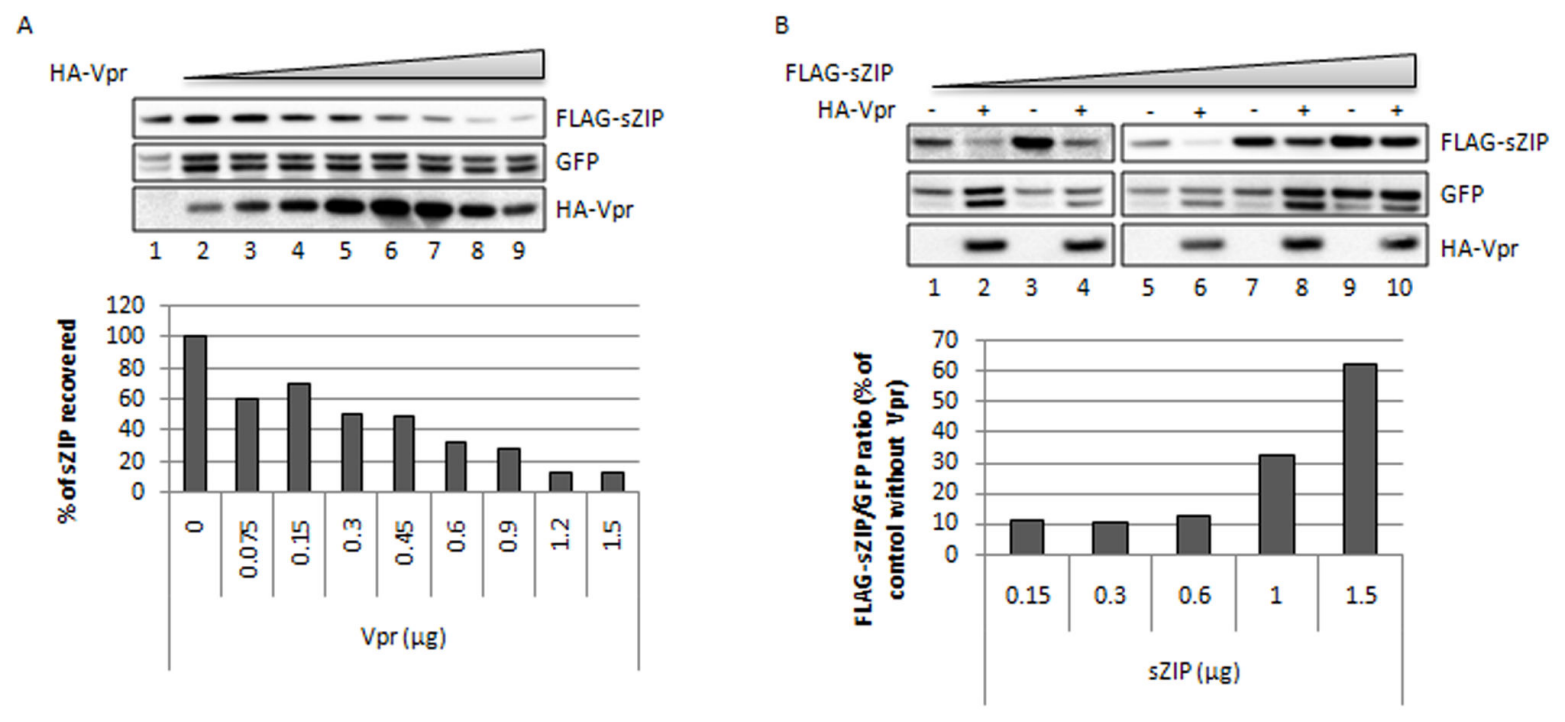
HIV-1 Vpr decreases the expression of sZIP in a dose-dependent manner. **A**. HeLa cells were co-transfected with a vector expressing FLAG-sZIP and with increasing amounts of a vector expressing HA-tagged Vpr. A GFP expression vector was used as an internal transfection control. Cells were harvested 48h post-transfection, lysed and protein expression analyzed by Western Blot (top panel). The histogram (bottom panel) displays the ratio between the FLAG signal and the GFP signal compared to this ratio without Vpr. B. Same as in A except with increasing amounts of the vector expressing FLAG-sZIP, with or without HA-tagged Vpr.

**Figure 3:**
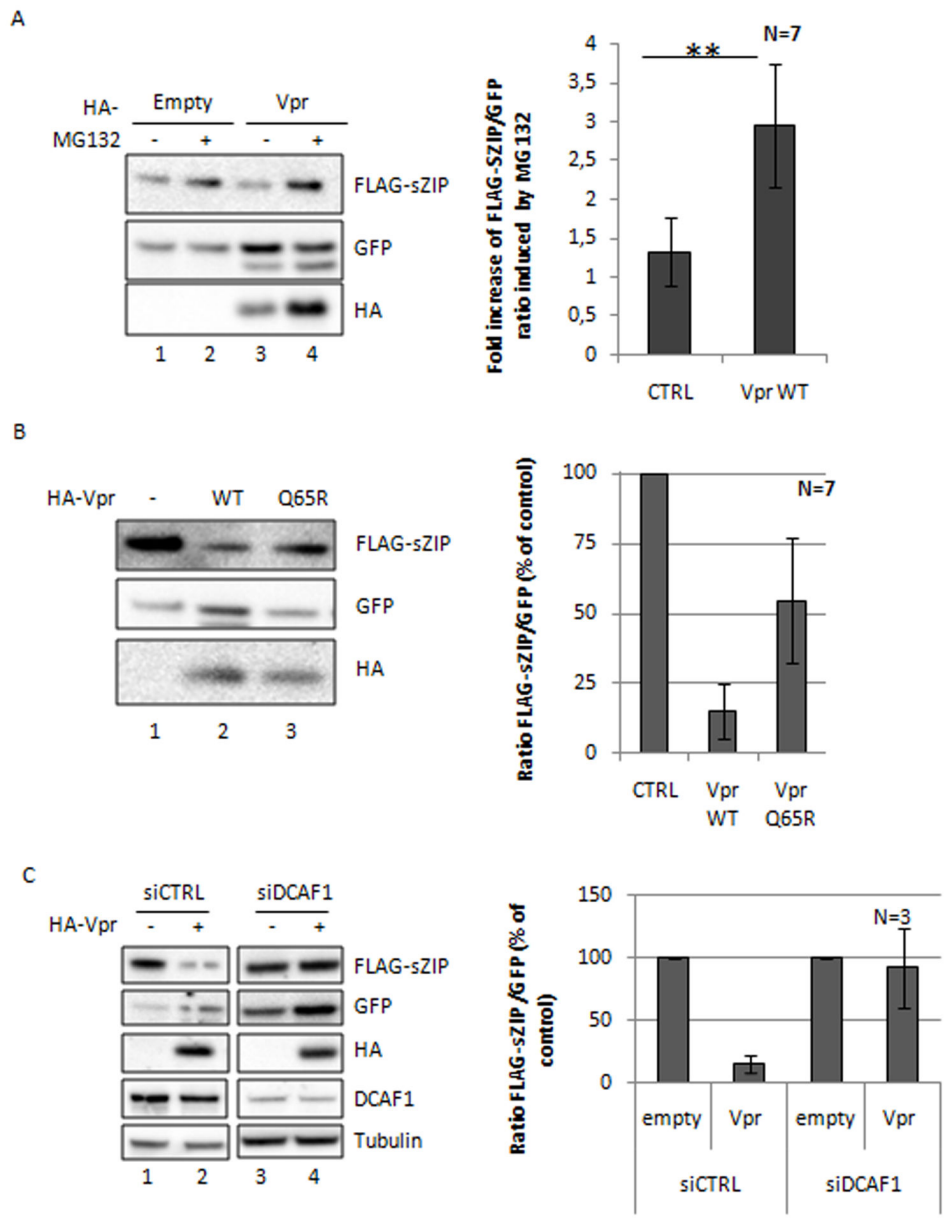
HIV-1 Vpr induces the degradation of sZIP through the DCAF1 ubiquitin ligase. **A**. Vpr-mediated sZIP degradation is dependent on the proteasome activity. HeLa cells were co-transfected with vectors expressing the indicated proteins. Cells were treated 48h post-transfection with or without 20µM MG132 for 6h, harvested and lysed. Proteins expression was analyzed by Western Blot. The left panel displays one representative experiment; the histogram shows the fold increase of sZIP expression (ratio over GFP) induced by MG132 with and without Vpr (7 independent experiments, p-value≈0.001). **B**. The DCAF1 binding-deficient Vpr mutant, VprQ65R, is less efficient than wt Vpr to induce sZIP degradation. HeLa cells were co-transfected with vectors expressing FLAG-sZIP, HA-tagged Vpr proteins as indicated and a GFP expression vector as an internal control (ratio 10:1). Cells were harvested 48h post-transfection, lysed and protein expression was analyzed by Western Blot (left panel, one representative experiment). The histogram shows the quantification of the ratio between the FLAG and GFP signals for 7 independent experiments. **C**. Silencing of DCAF1 impairs Vpr-induced sZIP degradation. HeLa cells were treated with either 50nM of control siRNA or with 50nM of siRNA directed against DCAF1. Cells were transfected 24h later with vectors expressing the indicated proteins. Cells were harvested 48h post-transfection, lysed and the proteins expression analyzed by Western Blot (left panel, one representative experiment). The histograms (right panel) display the ratios between FLAG and GFP signals.

We next investigated how the expression levels of ZIP or sZIP are affected by the presence of Vpr. Identical results were obtained with sZIP (main figures) and ZIP (supplementary figures). sZIP/ZIP was co-expressed with GFP, as an internal control, in HeLa cells in the presence or absence of Vpr. After cell harvesting and western blot analysis, ratios of sZIP/GFP (or ZIP/GFP) were determined. Vpr induced a drastic decrease of sZIP or ZIP expression in a dose-dependent manner (Figure 2A and Figure S3A respectively). Conversely, increasing the amounts of sZIP or ZIP overcame Vpr-mediated reduction of their expression indicating that the relative ratio of sZIP or ZIP to Vpr determines degradation of sZIP/ZIP (Figure 2B and Figure S3B respectively). Thereafter, we will refer to "degrading conditions" (use of ratios that favor Vpr-mediated ZIP/sZIP degradation) or "non-degrading conditions" (use of ratios that do not lead to ZIP/sZIP degradation in the presence of Vpr). Subsequent experiments were performed in "degrading conditions" leading to about 85% decrease of sZIP or ZIP expression. Proteasome inhibition by MG132 led to the reversion of Vpr-mediated inhibition of sZIP expression (Figure 3A, data not shown for ZIP), suggesting that Vpr induced the degradation of sZIP and ZIP in a proteasome-dependent manner. Overexpression of the VprQ65R mutant, which is impaired in its ability to recruit the DCAF1 ubiquitin ligase, did not reduce sZIP (or ZIP) expression as efficiently as wt Vpr (about 50% of sZIP recovered with VprQ65R compared to less than 15% for wt Vpr, Figure 3B). The remaining effect of this mutant on sZIP (or ZIP) expression is likely due to residual binding to DCAF1 (data not shown). Moreover silencing of DCAF1 completely abolished Vpr-mediated degradation of sZIP (compare lanes 2 and 4, Figure 3C) or ZIP (compare lanes 2 and 4, Figure S3C). Altogether, these data strongly suggest that Vpr hijacks the DCAF1 ubiquitin ligase to induce the degradation of sZIP and ZIP.

### Vpr-mediated sZIP/ZIP degradation is not involved in the cell growth-related Vpr activities

**Figure 4:**
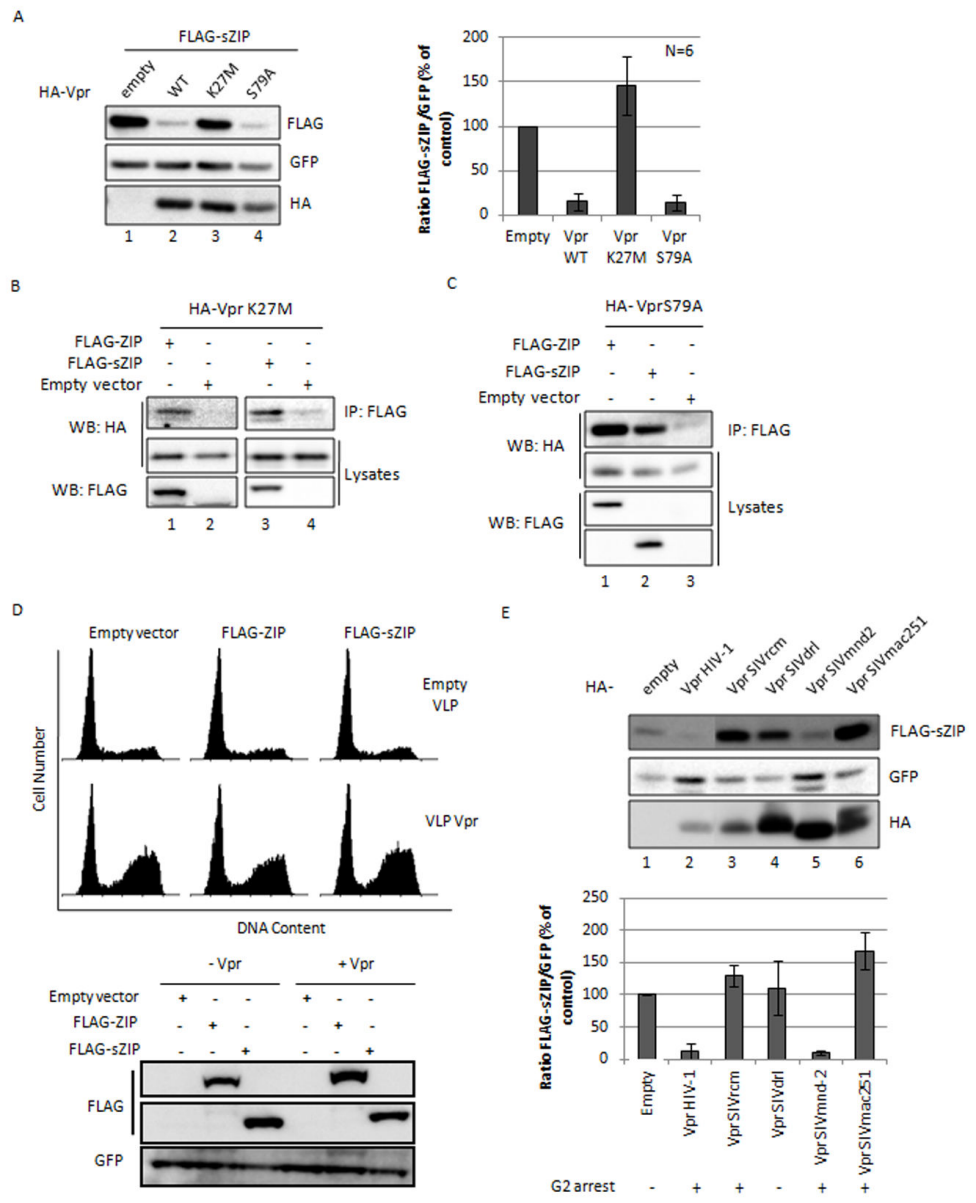
Vpr-mediated sZIP degradation does not correlate with the G2 arrest-independent cytotoxicity activity of Vpr, nor with its ability to trigger G2 arrest. **A**. Characterization of Vpr mutants for their ability to trigger the degradation of sZIP. HeLa cells were co-transfected with vectors expressing FLAG-sZIP and the indicated HA-tagged Vpr proteins and a GFP expression vector as an internal control (ratio 10:1). Cells were harvested 48h post-transfection, lysed and proteins expression was analysed by Western Blot. The top panel displays the results of one representative experiment. The bottom panel shows the quantification of the ratio between FLAG and GFP signals for several independent experiments. **B** and **C**. G2 arrest-defective Vpr mutants, VprK27M and VprS79A, still interact with ZIP and sZIP. HEK293T cells were transfected with vectors expressing HA-tagged Vpr mutants, VprK27M (**B**) and VprS79A (**C**), and a vector expressing the indicated FLAG-tagged proteins. Cell lysates were prepared 48h post-transfection and subjected to immunoprecipitation using anti-FLAG antibodies as described in Figure 1B. **D**. Overexpression of ZIP or sZIP does not overcome Vpr-mediated G2 arrest. HeLa cells were co-transfected with vectors expressing either FLAG-ZIP or FLAG-sZIP along with a vector expressing the GFP protein. 24h post-transfection, the cells were incubated with empty VLP or VLP containing wt Vpr protein for 2 h (800 ng GAG CAp24 per VLP). The cells were harvested 18h after the VLP treatment. Half the cells were fixed, stained with propidium iodide and analyzed by flow cytometry to monitor the DNA content of the GFP-positive population (top panel). The other half of the cells were lysed and protein expression was analyzed by Western Blot (bottom panel). **E**.The Vpr-induced sZIP degradation has some Vpr-species specificity (which does not correlate with Vpr-species specificity towards cell cycle arrest). HeLa cells were co-transfected with a vector expressing FLAG-sZIP together with a vector expressing the indicated HA-tagged Vpr proteins. GFP was used as an internal control as in A. Cells were harvested 48h post-transfection, lysed and protein expression was analyzed by Western Blot (top panel). The bottom panel shows the ratios between FLAG and GFP signals. The G2 arrest activity of each Vpr protein in Hela cells is indicated below the histogram.

We further investigated whether the two cell growth-related Vpr activities (G2 arrest and G2 arrest-independent cytotoxicity) could result from Vpr-mediated degradation of sZIP or ZIP. To this aim, we used previously characterized Vpr mutants and tempted to correlate their functional phenotype with their ability to degrade sZIP or ZIP. We first studied the phenotype of the VprK27M mutant, which does not arrest the cell cycle at the G2 phase but is still cytotoxic in a G2 arrest-independent manner [26]. This mutant failed to induce the degradation of ZIP and sZIP (Figure 4A and Figure S4A, compare lanes 1 to 3) but still interacted with both proteins (Figure 4B). Thus sZIP and ZIP are most likely not the cellular factors targeted by Vpr to trigger G2 arrest-independent cell death. A second mutant, VprS79A, which has lost its ability to arrest the cell cycle in G2 [15,18], still interacted with both sZIP and ZIP *in vivo* (Figure 4C) and induced their degradation (Figure 4A and Figure S4A, compare lanes 1, 2 and 4). This result suggests that sZIP and ZIP are not involved in the cytostatic activity of Vpr, or at least that their degradation is not sufficient for Vpr-mediated G2 arrest. We postulated that if the degradation of ZIP or sZIP was required for the cytostatic activity of Vpr, their overexpression would abolish Vpr-mediated G2 arrest. We conducted a cell cycle experiment in which exogenous ZIP and sZIP were expressed in HeLa cells one day prior to the delivery of Vpr with virus-like-particles (VLPs). Cell cycle analysis was carried out 18 hours after Vpr delivery. In these "non-degrading conditions" (see western blot Figure 4D, bottom panel), the presence of ZIP or sZIP did not affect Vpr-mediated G2 arrest (Figure 4D, upper panel). This result further indicates that ZIP and sZIP are not involved in the cytostatic activity of Vpr. We also analyzed the ability of Vpr proteins from distinct lentiviral lineages to induce the degradation of sZIP or ZIP. Vpr from SIVmnd-2, which is able to induce G2 arrest ( [26] and Figure S5), mediated the degradation of both isoforms as efficiently as Vpr from HIV-1 (Figure 4E and Figure S4B, compare lanes 1, 2 and 5) and Vpr from SIVdrl, which does not induce G2 arrest (Figure S4C), failed to induce the degradation of sZIP and ZIP (Figure 4E and Figure S4B). However, despite their ability to arrest the cell cycle [26,39], the Vpr proteins from SIVrcm and SIVmac251 failed to induce the degradation of ZIP or sZIP. Therefore, the ability of Vpr to induce sZIP or ZIP degradation did not correlate with its ability to induce cell cycle arrest in human cells. Altogether, our results discard sZIP and ZIP as the cellular factors targeted by Vpr to induce cell cycle arrest at the G2 phase.

In an attempt to understand the role of ZIP and sZIP in the viral life cycle, we wondered whether the two proteins could interfere with HIV-1 LTR-driven viral transcription. HeLa cells were transfected with increasing doses of vectors expressing ZIP or sZIP, then infected with a VSV-G pseudotyped HIV-1 virus expressing luciferase (pNL4.3LucΔEnvΔVpr). Quantification of luciferase showed that ZIP and sZIP failed to repress or activate LTR-driven transcription in HeLa cells (Figure S5A).

**Figure 5:**
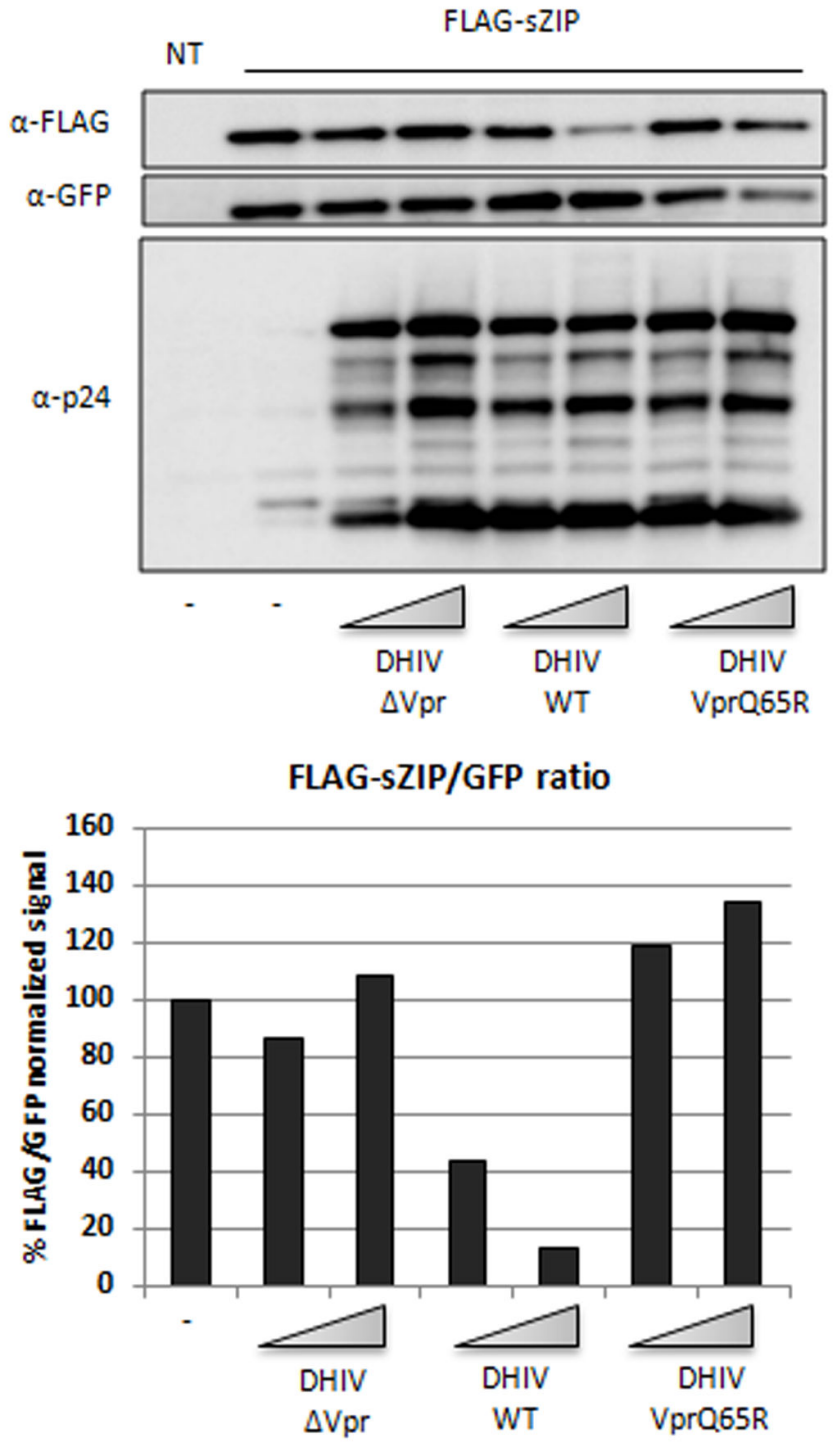
Vpr expressed following infection with HIV-1 reduces exogenous sZIP expression levels. 293T cells were co-transfected with equal amounts of empty or FLAG-sZIP-expressing plasmid in the presence of a GFP expression vector as an internal transfection control (ratio 10:1). After 24h the cells were mock infected or infected with two doses of the indicated HIV-1 viruses (50 and 250 ng of GAG CAp24 per 10^5^ cells). Two days post-infection the cells were lysed and expression levels of FLAG-sZIP, GFP and GAG products were assessed by western-blot in the whole cell extracts (top panel). The histogram (bottom panel) displays the FLAG/GFP signal ratios.

We further asked whether the two transcription factors could be degraded in the context of viral infection. HEK293 cells were first transfected with vectors encoding ZIP and sZIP, then infected with HIV-1 viruses lacking or encoding the Vpr gene or bearing the Vpr gene mutated at the Q65 residue. Expression of sZIP and ZIP was reduced in the presence of wt viruses but not when Vpr was deleted or mutated on its DCAF1 binding site (Figures 5 and S5B).

## Discussion

We uncovered ZIP as a new HIV-1 Vpr-interacting partner with a high confidence score in a two-hybrid screen. We considered ZIP and its isoform sZIP as potential Vpr targets for several reasons: (i) Vpr binds to chromatin, (ii) ZIP and sZIP recruit the NuRD chromatin remodeling complex and (iii) these two proteins play a role in cell proliferation and survival. We found that Vpr induced ZIP and sZIP degradation through the use of the DCAF1 adaptor of the Cul4A^DDB1^ ubiquitin ligase. The physiological relevance of this phenomenon regarding HIV-1 remains uncertain. However, degradation of overexpressed ZIP or sZIP also occurred in the context of infection by HIV-1 viruses bearing a wt Vpr gene and not the corresponding DCAF1-binding deficient mutant.

Previous reports have shown that Vpr associates with chromatin, though the percentage of Vpr bound to the chromatin fraction was not determined [22,23,40,41]. Importantly, biochemical and immunofluorescence approaches showed that Vpr could form a complex with DCAF1 on chromatin [22]. In addition, Cul4A^DDB1^ is an ubiquitin ligase specialized in the degradation of substrates present in the vicinity of the chromatin [42]. The finding that the chromatin fraction is the only one in which Vpr, DCAF1, DDB1 and Cul4A are present together nicely fits with these data, reinforcing the idea, previously raised by Belzile et al., that Vpr may induce the degradation of cellular targets on the chromatin [22]. Nonetheless, in our hands most of the nuclear portion of Vpr was soluble and only less than 3% of the viral protein was tightly bound to the chromatin. This poor recruitment of Vpr to chromatin could result from our experimental procedure, in which the chromatin fraction contains proteins only tightly bound to chromatin. In any case, a small amount of the viral protein should be sufficient to ensure its activities through an ubiquitin ligase enzymatic activity. In a similar way, Precious et al. underscored a catalytic process, where a small pool of SV5 V protein is recycled in order to degrade a large excess of STAT1 through the use of Cul4A^DDB1^ [43].

Our interest in NuRD was driven by the fact that both ZIP and sZIP were shown to recruit this chromatin remodeling complex [32,33]. Interestingly, RbAp46, HDAC1 and MTA2 on the one hand and ZIP and sZIP expressed from transfected vectors on the other hand were present in the chromatin fraction. Of note, Vpr interacted with RbAp46, weakly with MTA2 but not with every constituent of the NuRD complex and namely not with HDAC1. Unfortunately, no Vpr mutant, defective for ZIP or sZIP interaction, could be isolated so far. Altogether, our data suggest that Vpr might be present within a sub-complex, containing ZIP (or sZIP), RbAp46 and HAT1 (Figure 1F, dotted-line box).

The dose-dependent effect we have noticed regarding Vpr-mediated ZIP or sZIP degradation was also reported in the case of UNG2 [27]. In both examples, the ratio of Vpr over the cellular target is critical for degradation to occur. Different physiological outcomes may result from the interaction depending on this ratio. Further work is required to determine the relative expression of ZIP and sZIP in primary cells relevant for HIV infection.

Over the past years, many cellular partners of Vpr have been identified and new interactions are still being unravelled. The relevance of these interactions is sometimes difficult to assess. Studying the role of Vpr-ZIP (or sZIP) interaction suffers from a lack of a clear *in vitro* culture system where HIV-1 replication would show a strong dependence on the presence of the viral protein. Indeed, it is only in such a system that the cellular target inactivated by Vpr is expected to display an antiviral activity. Similarly, specific cellular systems are essential to highlight the activity of cellular restriction factors against HIV. In these cells, the restriction factor is both present and active. For example, the antiviral activity of the SAMHD1 restriction factor can only be unravelled in quiescent cells, where its viral counterpart, Vpx, enhances HIV-1 transduction [2,7,8]. In a system where the activity of Vpr could be easily monitored, suppression of the expression of ZIP or sZIP by siRNA would be a valuable tool to demonstrate a potential antiviral activity associated with these cellular proteins.

The situation is even more complicated with Vpr since Vpr likely uses the same ubiquitin ligase, Cul4A^DDB1^, to inactivate several proteins that may display an antiviral activity in different settings. Indeed, on the one hand, we have provided genetic evidence that Vpr hijacks Cul4A^DDB1^ to arrest the cell cycle and to induce a G2 arrest-independent cytotoxicity phenotype [18,26]. On the other hand, Vpr has been reported to use Cul4A^DDB1^ to inactivate several distinct proteins, namely Mfn2, Ung2 and Dicer [25,44,45]. Similarly, the E6 protein from papillomavirus targets a wide range of cellular proteins for proteasome-mediated degradation, including p53, the Bcl-2 family member Bak and many other targets containing class 1 PDZ domains including Akt, Dlg and Scribble (for a review, see [46]).

Cellular proteins inactivated by Vpr might share some structural or functional features. Keeping in mind that (i) UNG2 is a glycosylase that removes uracil near replication forks; (ii) that the G2 target is thought to be present in the vicinity of the chromatin [22]; (iii) that ZIP/sZIP are NuRD binding partners and (iv) that Cul4A and DDB1 are present mostly in the chromatin fraction, a common feature of the Vpr targets might be their association with chromatin, at least when degradation occurs.

Vpr-mediated ZIP or sZIP degradation correlated neither with the most studied property of Vpr, i.e. its ability to induce G2 arrest, nor with its G2 arrest-independent cytotoxic property. Interaction of Vpr with ZIP or sZIP could be involved in some other Vpr activity, namely its ability to trans-activate the LTR or to modulate transcription from cellular promoters [47-58]. We have not investigated this hypothesis yet because of the lack of Vpr mutants for such activities. In addition, we found that overexpression of ZIP or sZIP did not affect transcription driven from the HIV-1 promoter in HeLa cells. Future work will be needed to assess whether Vpr interferes with the transcriptional activities of ZIP and sZIP in order to provide an advantageous environment for viral replication and dissemination. A comparative transcriptional profiling of cells expressing or not Vpr could help gain some insight into the function of Vpr.

## Materials and Methods

### Plasmid constructs.

pAS1B vectors encoding HA-tagged Vpr from HIV-1 LAI, Vpr from SIVrcm, Vpr from SIVmnd2 and Vpr from SIVmac251 have been previously described [18,26,59]. The Vpr gene corresponding to SIVdrl was synthesized by GeneCust Europe following codon optimization for expression in human cells and then inserted into the pAS1B vector. Plasmids encoding ZIP and sZIP fused to the FLAG tag at the N-terminus have been previously described [32,33]. The internal membrane-anchored GFP was expressed from the pBabe/GEM2 vector [60].

### Yeast Two-Hybrid analysis

The coding sequence for full-length YU2 VPR (nt5557 to nt 5850) was PCR-amplified and cloned into pB27 as a C-terminal fusion to LexA. The constructs were used as baits to screen at saturation a highly complex dT-primed human CEMC7 library. 60.3 million clones (6-fold the complexity of the library) were screened using a mating approach with Y187 (mata) and L40DGal4 (mata) yeast strains as previously described [61]. His+ LacZ+ colonies were grown on a medium lacking tryptophan, leucine, and histidine. 265 colonies were selected. The prey fragments of the positive clones were amplified by PCR and sequenced at their 5p junction. The resulting sequences were used to identify the corresponding interacting proteins in the GenBank database (NCBI), using a fully automated procedure. Briefly, 5p sequences were filtered using PHRED. Sequence contigs were built using CAP3 and compared to the latest release of GenBank using BLASTN [62]. 14 clones coding for ZIP were selected with 8 different fusions.

### Cell culture, transfection procedures and reagents

HeLa and HEK293T cells were maintained in DMEM supplemented with glutamine and 10% fetal calf serum. Plasmid transfections were performed using Fugene 6 Transfection reagent (Roche) and siRNA transfections were performed using Dharmafect reagent (Dharmacon). Control and DCAF1 siRNAs were purchased from Dharmacon. The following siRNA was used to target DCAF1: 09, ggagggaaUUgucgagaauuu. The proteasome inhibitor MG132 was purchased from Sigma and used at a final concentration of 20µM for 6 hours.

### Subcellular fractionation assay

The subcellular fractionation was carried out using the “Subcellular Protein Fractionation” kit from Pierce according to the manufacturer’s instructions. Briefly, 2x10^6^ HeLa cells were lyzed with successive buffers, in the presence of protease inhibitors, allowing the successive extraction of soluble cytoplasmic proteins, membrane-bound proteins, soluble nuclear proteins and then chromatin-bound proteins (issued from micrococcal nuclease digestion), leaving insoluble proteins in the remaining pellet. The ratio of the final volume of each fraction is 2:2:1:1:1.

### Anti-FLAG immunoprecipitation procedure

Cells grown in 10-cm dishes were lyzed in 700µl SD buffer (50mM Tris‑HCl pH 7.5, 150mM NaCl, 0.5% Triton X100) containing an anti-protease cocktail (Sigma). Cell lysates were clarified by centrifugation and incubated with anti-FLAG beads (EZview^™^ Red ANTI-FLAG^®^ M2 Affinity Gel, Sigma) overnight at 4°C. After four washes in SD buffer, immunoprecipitated proteins were recovered by elution with Flag peptide (Sigma) for one hour at 4°C.

### Anti-HA immunoprecipitation procedure

Cells grown in 10-cm dishes were lyzed in 700µl SD buffer containing an anti-protease cocktail (Sigma). Cell lysates were clarified by centrifugation and incubated with anti-HA beads (Anti-HA Affinity Matrix, Roche) over-night at 4°C. After four washes in SD buffer, immunoprecipitated proteins were recovered by elution with HA peptide (Roche) for one hour at 37°C.

### Western blot procedure and antibodies

Cells were lyzed in 500µl SB buffer (60mM Tris pH 8, 10% Glycerol, 2% SDS, 1% bromophenol blue, 100mM DTT) using a 27G needle and protein extracts were separated by SDS-PAGE electrophoresis. Following transfer onto PVDF membranes, proteins were revealed by immunoblot analysis using a chemiluminescent procedure (CDP*Star*®, Applied Biosystems). Signals were acquired by a LAS 3000 apparatus (Fujifilm) for further quantification, using the Multigauge software (Fujifilm). Monoclonal antibody directed against the HA (16B12) tag was purchased from Covance Research Products; anti-GFP from Roche; anti-FLAG M2 and anti-Tubulin monoclonal antibodies from Sigma, anti-Cullin4A and RbAp46 rabbit polyclonal antibodies and anti-MTA2 mouse monoclonal antibody from Abcam; anti-Histone H4 and anti-HDAC1 rabbit polyclonal antibodies were purchased from Cell Signaling; anti-DCAF1 rabbit polyclonal antibody was obtained from Gentaur; anti-DDB1 mouse monoclonal antibody was purchased from Zymed and anti-HAT1 mouse monoclonal antibody was purchased from Santa Cruz.

### Virus production

For the delta-Env HIV-1 viruses (DHIV), 293T cells (4 × 10^6^ cells) were co-transfected with pNL4.3 deltaEnv HIV-1 constructs lacking the gene encoding Vpr (DHIV ΔVpr) or encoding either wt Vpr (DHIV wt) or VprQ65R (DHIV VprQ65R) [63] along with a plasmid encoding the vesicular stomatitis virus glycoprotein G (VSV-G). For the luciferase reporter viruses, 293T cells were co-transfected with a pNL4.3 deltaEnv-deltaVpr HIV-1 construct expressing the luciferase protein instead of Nef [10] along with the plasmid encoding VSV-G. For the pseudoparticles (VLPs), 293T cells were cotransfected with HIV-1 minimal packaging vector pCMVdelta8.91 [11] along with the plasmid encoding VSV-G and a plasmid encoding HA-tagged Vpr in a ratio of 5:1:5. The culture supernatants were collected 48h after transfection and filtered through 0.45-µm pore filters. The viral particles were then concentrated in 10% polyethylene-glycol 6000 (PEG-6000) (Sigma) containing 300mM NaCl and titrated by quantification of HIV-1 capsid p24 using an enzyme-linked immunosorbent assay (ELISA) (ZeptoMetrix Corporation).

### Luciferase activity assay

The luciferase activity was measured using the Dual-Luciferase® Reporter Assay from Promega according to the manufacturer’s instructions. Briefly, cells expressing luciferase were washed twice with PBS and lysed directly in wells using 1X Passive Lysis Buffer for 15 minutes at room temperature. Cell lysates were clarified by centrifugation and the luciferase activity was measured using Luciferase Assay Substrate with a FLUOstar OPTIMA from BMG LABTECH.

### Cell cycle analysis

5x10^5^ HeLa cells were plated onto 6-cm dishes 24 hours prior to transfection. The cells were transfected with 1 µg of pAS1B-Vpr in combination with 0.1 µg of pBabe/GEM2 as an internal transfection marker. Twenty-four hours later, the cells were harvested and plated onto 10-cm dishes and grown for one more day. The cells were then detached (manually) and fixed in 70% ethanol. Following treatment for 30 minutes at 37°C with 0.2 mg/ml RNase A and 50 µg/ml propidium iodide in buffer H (20 mM Hepes, 160 mM NaCl, 1 mM EGTA), cells expressing the cotransfected GFP were analyzed for their DNA content using a Cytomics FC500 cell analyzer (Beckman Coulter). At least 10,000 GFP-positive cells were analyzed for their distribution in the different phases of the cell cycle.

## Supporting Information

Figure S1 **Only a small fraction of Vpr is detected in the chromatin-associated proteins fraction.** The histogram displays the quantification of HA signal in each fraction, expressed as a percentage of the total HA signal for cells expressing HA-Vpr WT from the Western blot displayed in Figure 1C.
(TIF)

Figure S2 **ZIP and sZIP interact with subunits of the Mi2/NuRD complex and with HAT1, a partner of RbAp46.** HEK293T cells were transfected with vectors expressing HA-tagged Vpr and the indicated FLAG-tagged proteins. Cell lysates were prepared 48h post-transfection and subjected to immunoprecipitation using anti-FLAG antibodies. After extensive washing, bound proteins were eluted from beads with a FLAG peptide. Immunoprecipitates (*IP*) and crude cell lysates (*Lysates*) were analyzed by Western blotting using the indicated antibodies.
(TIF)

Figure S3 **HIV-1 Vpr induces the degradation of ZIP through the DCAF1 ubiquitin ligase.** **A** and **B**. HIV-1 Vpr decreases the expression of ZIP in a dose-dependent manner. **A**. HeLa cells were co-transfected with a vector expressing FLAG-ZIP and with increasing amounts of a vector expressing HA-tagged Vpr. A GFP expression vector was used as an internal transfection control. Cells were harvested 48h post-transfection, lysed and protein expression analyzed by Western Blot (top panel). The histogram (bottom panel) displays the ratio between the FLAG signal and the GFP signal compared to this ratio without Vpr. B. Same as in A except with increasing amounts of the vector expressing FLAG-ZIP, with or without HA-tagged Vpr. C. Silencing of DCAF1 impairs Vpr-induced ZIP degradation. HeLa cells were treated with either 50nM of control siRNA or with 50nM of siRNA directed against DCAF1. Cells were transfected 24h later with vectors expressing the indicated proteins. Cells were harvested 48h post-transfection, lysed and the proteins expression analyzed by Western Blot (left panel, one representative experiment). The histograms (right panel) display the ratios between FLAG and GFP signals.
(TIF)

Figure S4 **Vpr-mediated ZIP degradation does not correlate with the G2 arrest-independent cytotoxicity activity of Vpr, nor with its ability to trigger G2 arrest.** **A**. Characterization of Vpr mutants for their ability to trigger the degradation of ZIP. HeLa cells were co-transfected with vectors expressing FLAG-ZIP and the indicated HA-tagged Vpr proteins and a GFP expression vector as an internal control (ratio 10:1). Cells were harvested 48h post-transfection, lysed and proteins expression was analysed by Western Blot. The top panel displays the results of one representative experiment. The bottom panel shows the quantification of the ratio between FLAG and GFP signals for several independent experiments. **B**. The Vpr-induced ZIP degradation has some Vpr-species specificity (which does not correlate with Vpr-species specificity towards cell cycle arrest). HeLa cells were co-transfected with a vector expressing FLAG-ZIP together with a vector expressing the indicated HA-tagged Vpr proteins. GFP was used as an internal control as in A. Cells were harvested 48h post-transfection, lysed and protein expression was analyzed by Western Blot (top panel). The bottom panel shows the ratios between FLAG and GFP signals. The G2 arrest activity of each Vpr protein in Hela cells is indicated below the histogram. **C**. SIVdrl Vpr does not induce a cell cycle arrest at the G2/M transition. HeLa cells were transfected with vectors expressing the indicated HA-tagged proteins along with a vector expressing the GFP protein. Cells were harvested 48 h post-transfection. After fixation and propidium iodide staining, the cells were analyzed by flow cytometry to monitor the DNA content of the GFP-positive population. The G2/G1 ratio is indicated above each diagram.
(TIF)

Figure S5 **A. ZIP and sZIP do not affect transcription from the LTR promoter in HeLa cells.** HeLa cells were transfected with vectors expressing either FLAG-ZIP or FLAG-sZIP (1.5 and 3 µg of each). Cells were then infected 24h post-transfection with VSV-G pseudo-typed pNL4.3LucΔEnvΔVpr at MOI 0.5. Cells were harvested 48h post-infection, lysed and the luciferase activity was measured using a FLUOstar OPTIMA from BMG Labtech (AU, Arbitrary Units) (top panel). The experiment was performed in triplicate. Expression levels of FLAG-ZIP and FLAG-sZIP were determined by western blot (bottom panel.) **B**. **Vpr expressed following infection with HIV-1 decreases exogenous ZIP expression**. 293T cells were co-transfected with equal amounts of empty or FLAG-ZIP-expressing plasmid in the presence of a GFP expression vector. Twenty four hours post-transfection, cells were infected with two doses of the indicated HIV-1 viruses (50 and 250 ng of GAG CAp24 per 10^5^ cells). Two days post-infection the cells were lysed and expression levels of FLAG-ZIP, GFP and GAG products were assessed by western-blot in the whole cell extracts.
(TIF)
