# Supplementary figures and images for: HIV-1 Vpr Induces the Degradation of ZIP and sZIP, Adaptors of the NuRD Chromatin Remodeling Complex, by Hijacking DCAF1/VprBP
Source: PLoS One. 2013 Oct 8;8(10):e77320. doi: 10.1371/journal.pone.0077320 (PMC3792905; doi:10.1371/journal.pone.0077320)

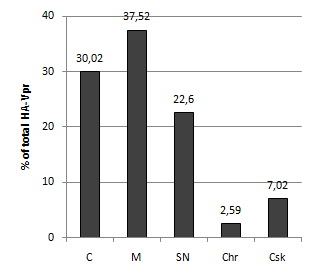

Supplement: Figure S1 — Only a small fraction of Vpr is detected in the chromatin-associated proteins fraction. The histogram displays the quantification of HA signal in each fraction, expressed as a percentage of the total HA signal for cells expressing HA-Vpr WT from the Western blot displayed in Figure 1C. (TIF) [file pone.0077320.s001.tif]

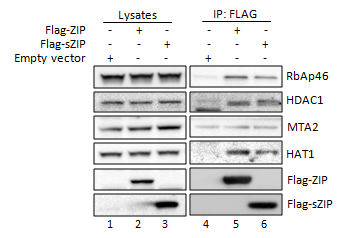

Supplement: Figure S2 — ZIP and sZIP interact with subunits of the Mi2/NuRD complex and with HAT1, a partner of RbAp46. HEK293T cells were transfected with vectors expressing HA-tagged Vpr and the indicated FLAG-tagged proteins. Cell lysates were prepared 48h post-transfection and subjected to immunoprecipitation using anti-FLAG antibodies. After extensive washing, bound proteins were eluted from beads with a FLAG peptide. Immunoprecipitates (IP) and crude cell lysates (Lysates) were analyzed by Western blotting using the indicated antibodies. (TIF) [file pone.0077320.s002.tif]

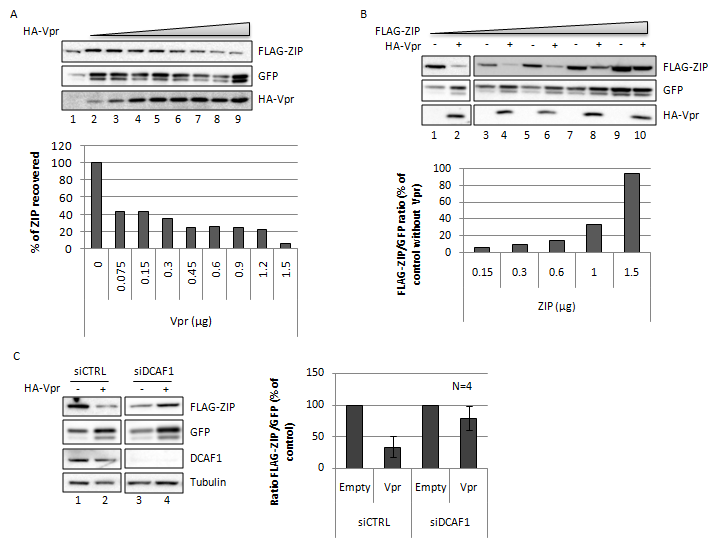

Supplement: Figure S3 — HIV-1 Vpr induces the degradation of ZIP through the DCAF1 ubiquitin ligase. A and B. HIV-1 Vpr decreases the expression of ZIP in a dose-dependent manner. A. HeLa cells were co-transfected with a vector expressing FLAG-ZIP and with increasing amounts of a vector expressing HA-tagged Vpr. A GFP expression vector was used as an internal transfection control. Cells were harvested 48h post-transfection, lysed and protein expression analyzed by Western Blot (top panel). The histogram (bottom panel) displays the ratio between the FLAG signal and the GFP signal compared to this ratio without Vpr. B. Same as in A except with increasing amounts of the vector expressing FLAG-ZIP, with or without HA-tagged Vpr. C. Silencing of DCAF1 impairs Vpr-induced ZIP degradation. HeLa cells were treated with either 50nM of control siRNA or with 50nM of siRNA directed against DCAF1. Cells were transfected 24h later with vectors expressing the indicated proteins. Cells were harvested 48h post-transfection, lysed and the proteins expression analyzed by Western Blot (left panel, one representative experiment). The histograms (right panel) display the ratios between FLAG and GFP signals. (TIF) [file pone.0077320.s003.tif]

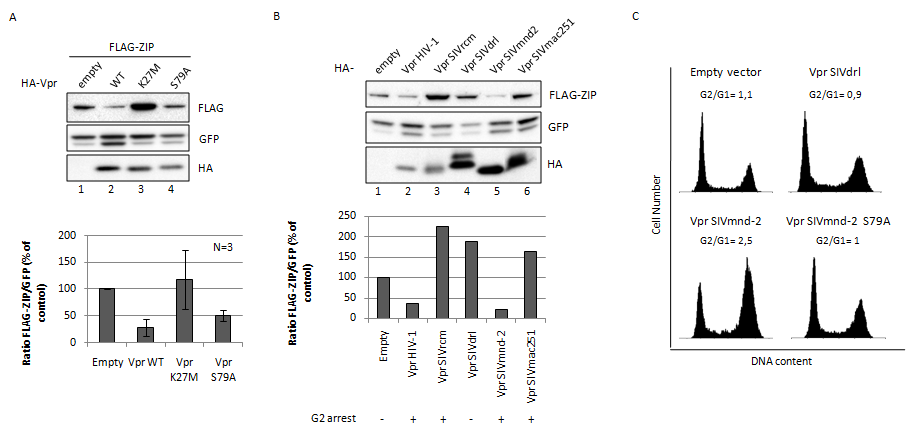

Supplement: Figure S4 — Vpr-mediated ZIP degradation does not correlate with the G2 arrest-independent cytotoxicity activity of Vpr, nor with its ability to trigger G2 arrest. A. Characterization of Vpr mutants for their ability to trigger the degradation of ZIP. HeLa cells were co-transfected with vectors expressing FLAG-ZIP and the indicated HA-tagged Vpr proteins and a GFP expression vector as an internal control (ratio 10:1). Cells were harvested 48h post-transfection, lysed and proteins expression was analysed by Western Blot. The top panel displays the results of one representative experiment. The bottom panel shows the quantification of the ratio between FLAG and GFP signals for several independent experiments. B. The Vpr-induced ZIP degradation has some Vpr-species specificity (which does not correlate with Vpr-species specificity towards cell cycle arrest). HeLa cells were co-transfected with a vector expressing FLAG-ZIP together with a vector expressing the indicated HA-tagged Vpr proteins. GFP was used as an internal control as in A. Cells were harvested 48h post-transfection, lysed and protein expression was analyzed by Western Blot (top panel). The bottom panel shows the ratios between FLAG and GFP signals. The G2 arrest activity of each Vpr protein in Hela cells is indicated below the histogram. C. SIVdrl Vpr does not induce a cell cycle arrest at the G2/M transition. HeLa cells were transfected with vectors expressing the indicated HA-tagged proteins along with a vector expressing the GFP protein. Cells were harvested 48 h post-transfection. After fixation and propidium iodide staining, the cells were analyzed by flow cytometry to monitor the DNA content of the GFP-positive population. The G2/G1 ratio is indicated above each diagram. (TIF) [file pone.0077320.s004.tif]

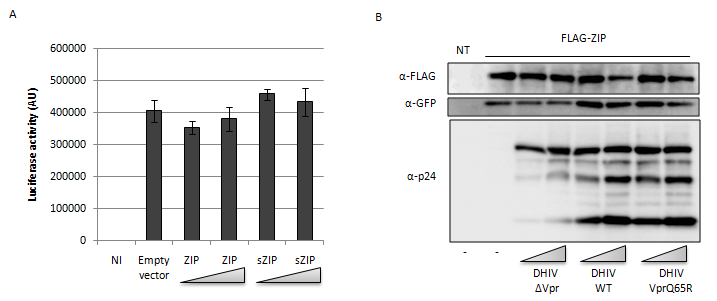

Supplement: Figure S5 — A. ZIP and sZIP do not affect transcription from the LTR promoter in HeLa cells. HeLa cells were transfected with vectors expressing either FLAG-ZIP or FLAG-sZIP (1.5 and 3 µg of each). Cells were then infected 24h post-transfection with VSV-G pseudo-typed pNL4.3LucΔEnvΔVpr at MOI 0.5. Cells were harvested 48h post-infection, lysed and the luciferase activity was measured using a FLUOstar OPTIMA from BMG Labtech (AU, Arbitrary Units) (top panel). The experiment was performed in triplicate. Expression levels of FLAG-ZIP and FLAG-sZIP were determined by western blot (bottom panel.) B. Vpr expressed following infection with HIV-1 decreases exogenous ZIP expression. 293T cells were co-transfected with equal amounts of empty or FLAG-ZIP-expressing plasmid in the presence of a GFP expression vector. Twenty four hours post-transfection, cells were infected with two doses of the indicated HIV-1 viruses (50 and 250 ng of GAG CAp24 per 105 cells). Two days post-infection the cells were lysed and expression levels of FLAG-ZIP, GFP and GAG products were assessed by western-blot in the whole cell extracts. (TIF) [file pone.0077320.s005.tif]
